# LANGUAGE USE, LITERACY SKILLS, AND ONLINE HEALTH INFORMATION-SEEKING IN LATER LIFE AMONG HISPANICS IN THE U.S.

**DOI:** 10.1093/geroni/igz038.989

**Published:** 2019-11-08

**Authors:** Roberto J Millar, Shalini Sahoo, Takashi Yamashita, Phyllis Cummins

**Affiliations:** 1 University of Maryland Baltimore County, Baltimore, Maryland, United States; 2 University of Maryland, Baltimore, Baltimore, Maryland, United States; 3 Scripps Gerontology Center, Miami University, Oxford, Ohio, United States

## Abstract

Online platforms, which are often in English, have become a common source of health information. Accordingly, language skills have been shown to be important for health information-seeking. In the United States, the use of online health information is generally low among Hispanics, particularly those with limited English proficiency. What is less clear, however, is how different measures of English proficiency may be linked to online health information seeking in later life. This study examines the associations between language spoken at home, literacy skills, and online health information seeking among middle age and older Hispanics in the U.S. Data of Hispanic adults aged 40 years and older (n = 315) come from the 2012/2014 Program for International Assessment of Adult Competencies (PIAAC). We used binary logistic regression models with complex sampling weights to examine online health information seeking as a function of primary language use at home (Spanish vs. English) and literacy skill assessment scores (low – high: 0 – 500 points). Results indicated that speaking Spanish at home (OR = 0.317, p < 0.05) is a negative predictor, and greater literacy skills (OR = 1.011, p < 0.05) is a positive predictor of online health information seeking. Findings from this study clarify possible health information disadvantages by limited English proficiency and lower literacy skills. We discuss how the impact of primary language use and literacy skills should be incorporated into future health communication and policy initiatives to address the barriers to health information among middle-aged and older Hispanic adults.

